# Supplementary material for: Multimodal communication and audience directedness in the greeting behaviour of semi-captive African savannah elephants
Source: Commun Biol. 2024 May 9;7:472. doi: 10.1038/s42003-024-06133-5 (PMC11082179; doi:10.1038/s42003-024-06133-5)
Supplement: Supplementary file 2 — Supplementary Information [file 42003_2024_6133_MOESM2_ESM.pdf]

Supplementary Tables and Figures

Supplementary Table 1. Pairs of subjects with average Nearest-Neighbour index values (NN_{AB}) and demographic information.

Subject 1	Subject 2	Average NN index
Doma (46, ♂)	Hwange (20, ♀)	0.157
Doma (46, ♂)	Kariba (20, ♀)	0.220
Doma (46, ♂)	Mainos (26, ♂)	0.161
Doma (46, ♂)	Tatu (40, ♀)	0.149
Hwange (20, ♀)	Kariba (20, ♀)	0.375
Mainos (26, ♂)	Tatu (40, ♀)	0.178
Masuwe (20, ♀)	Hwange (20, ♀)	0.122
Masuwe (20, ♀)	Kariba (20, ♀)	0.137

Supplementary Table 2. List of communication, vocalisation, and body act variables coded for each video.

Variable	Description
**Communication number**	The number of the communication being coded (e.g., VEC0001).
Date	The date when the data was collected.
File name	The synchronised file name being coded.
Signaller	The ID of the signaller.
Signaller age	The age of the signaller.
Signaller olfactory signals	Marks if the signaller produces any olfactory behaviours during the communication (e.g., urinating, defecating, secreting from temporal glands).
Recipient	The ID of the recipient.
Recipient age	The age of the recipient.
Signaller prior context	The behavioural context of the signaller before the communication started.
Signaller post context	The behavioural context of the signaller after the communication ended.
Communication Comment	Any comment about the communication or video that may be of interest.
**Vocalisation record**	The vocalisation type produced by the signaller (e.g., Rumble, Trumpet).
Vocalisation record number	The number of the vocalisation record being coded (e.g., VEV0001)
Signaller in frame	Marks whether the signaller was in frame when he/she produces the vocalisation.
Mouth open	Marks if the elephant keeps the mouth open when rumbling.
Combination	Marks whether the vocalisation is part of a combination with other calls.
Vocalisation duration analysis	Indicates how the vocalisation duration should be treated in the analysis (e.g., start excluded, end excluded etc).
Vocalisation Comment	Any comment about the vocalisation that may be of interest.
**Body act record**	The body act type produced by the signaller (e.g., Ear-Slap)
Body act record number	The number of the body act record being coded (e.g., VEG0001)

Gaze signaller	Marks if the signaller looks at the recipient just before he starts producing the body act.
Distance recipient	The distance of the recipient from the signaller.
Recipient position	The position of the recipient just before the signaller starts producing the body act with respect to the signaller´s body axis (e.g., parallel, perpendicular).
Body part	The body part used to produce the body act (e.g., ear, trunk)
Contact part	The body part of the signaller a body act makes contact with.
Repetition	Marks the number of repetitions for body acts consisting in the repetition of a single movement.
Laterality	The laterality of the body part used to produce the body act (e.g., left, right)
Recipient body part	The body part of the recipient the body act makes contact with or is directed to (e.g., mouth, genitals etc)
Object	Any object used to produce the body act (e.g., branch, tree).
Audible	Presence of audible sound produced by the body act (e.g., Ear-Flapping sound).
Directionality	Marks whether there is evidence that the body act directs the attention or behaviour of the recipient towards a location, object, elephant, movement.
Visual attention recipient	The state of visual attention of the recipient to the body act just before it is produced by the signaller.
Body act duration analysis	Indicates how the body act duration should be treated in the analysis (e.g., start excluded, end excluded etc).
Body act Comment	Any comment about the body act that may be of interest.

Supplementary Table 3. Olfactory behaviours used by the subjects during the *n*=89 greeting communications

Olfactory behaviours	n Communications	Frequency
Urinating	15	17%
Temporal gland secretion	16	18%
Urinating -Temporal gland secretion	31	35%
Urinating - Defecating - Temporal gland secretion	1	1%
None	21	24%
Unknown	5	6%
Total	89	

Supplementary Table 4. Frequencies of the use of body act types by the signallers when they were visually attending the recipient or not at the onset of their production. “Yes” indicates that the signaller was attending the recipient; “No” indicates that the signaller was not attending the recipient.

Body act type	Modality	Signaller visual attention	Frequency	Total	Percentage
Back-Towards	Silent-visual	Yes	15	18	0.833
		No	3	18	0.167
Ear-Brush	Tactile	Yes	5	5	1.000
		No	0	0	0.000
Ear-Flapping	Audible	Yes	245	270	0.907
		No	25	270	0.093
Ear-Slap	Audible	Yes	13	14	0.929
		No	1	14	0.071

Ear-Slight-Spread	Silent-visual	Yes	55	63	0.873
		No	8	63	0.127
Ear-Spread	Silent-visual	Yes	91	99	0.919
		No	8	99	0.081
Ears-Stiff	Silent-visual	Yes	135	148	0.912
		No	13	148	0.088
Head-Raise	Silent-visual	Yes	26	29	0.897
		No	3	29	0.103
Rump-Present	Silent-visual	Yes	15	18	0.833
		No	3	18	0.167
Rubbing-Other	Tactile	Yes	4	4	1.000
		No	0	4	0.000
Tail-on-Side	Olfactory?	Yes	41	51	0.804
		No	10	51	0.196
Tail-Raise	Olfactory?	Yes	43	47	0.915
		No	4	47	0.085
Tail-Stiff	Olfactory?	Yes	35	40	0.875
		No	5	40	0.125
Tail-Touch	Tactile	Yes	4	10	0.400
		No	6	10	0.600
Tail-Waggling	Olfactory?	Yes	90	105	0.857
		No	15	105	0.143
Trunk-Reach	Silent-visual	Yes	39	40	0.975
		No	1	40	0.025
Trunk-Reach_Touch_Unc	Unknown	Yes	10	10	1.000
		No	0	10	0.000
Trunk-Shaking	Silent-visual	Yes	12	15	0.800
		No	3	15	0.200
Trunk-Side-Swinging	Silent-visual	Yes	9	10	0.900
		No	1	10	0.100
Trunk-Swinging	Silent-visual	Yes	10	10	1.000
		No	0	10	0.000

Supplementary Table 5. Frequencies of the use of body act types by the signallers when the recipient was visually attending or not at the onset of their production. "Yes" indicates that the recipient was attending the body act; "No" indicates that the recipient was not attending the body act.

Body act type	Modality	Recipient visual attention	Frequency	Total	Percentage
Back-Towards	Silent-visual	Yes	14	18	0.778
		No	4	18	0.222
Ear-Brush	Tactile	Yes	4	5	0.800
		No	1	5	0.200
Ear-Flapping	Audible	Yes	221	266	0.831
		No	45	266	0.169
Ear-Slap	Audible	Yes	14	15	0.933
		No	1	15	0.067
Ear-Slight-Spread	Silent-visual	Yes	57	64	0.891
		No	7	64	0.109
Ear-Spread	Silent-visual	Yes	87	99	0.879
		No	12	99	0.121
Ears-Stiff	Silent-visual	Yes	122	138	0.884
		No	16	138	0.116
Head-Raise	Silent-visual	Yes	28	29	0.966
		No	1	29	0.034
Rump-Present	Silent-visual	Yes	18	18	1.000
		No	0	18	0.000
Rubbing-Other	Tactile	Yes	4	4	1.000
		No	0	4	0.000
Tail-on-Side	Olfactory?	Yes	22	41	0.537
		No	19	41	0.463
Tail-Raise	Olfactory?	Yes	16	32	0.500
		No	16	32	0.500
Tail-Stiff	Olfactory?	Yes	16	27	0.593
		No	11	27	0.407
Tail-Touch	Tactile	Yes	3	10	0.300
		No	7	10	0.700
Tail-Waggling	Olfactory?	Yes	36	62	0.581
		No	26	62	0.419
Trunk-Reach	Silent-visual	Yes	28	40	0.700
		No	12	40	0.300
Trunk-Reach_Touch_Unc	Unknown	Yes	7	9	0.778
		No	2	9	0.222

Trunk-Shaking	Silent-visual	Yes	10	14	0.714
		No	4	14	0.286
Trunk-Side-Swinging	Silent-visual	Yes	9	10	0.900
		No	1	10	0.100
Trunk-Swinging	Silent-visual	Yes	7	9	0.778
		No	2	9	0.222

Supplementary Table 6.
Results of GLMM exploring if the use of tail body acts by semi-captive elephants during greeting varies according to the visual attentional state of the recipient. *n*=543 body acts.

	estimate	*SE*	*Lwr CI*	*Upr CI*	χ^2	*P*	*min*	*max*
(Intercept)	0.349	0.335	-0.176	0.896		(1)	0.173	0,784
Recipient visual attention_Yes	-1.858	0.376	-2.456	-1.297	11.025	0.001	-2.199	-1.592

"Recipient visual attention" was dummy coded and centred before entering the random slope in the model. The table shows estimates, standard errors, bootstrapped confidence intervals, test results, and minimum and maximum of the model stability estimates after removing levels of random effects one at a time. Significant results are highlighted in bold. "(1)" Not indicated because of limited interpretation.

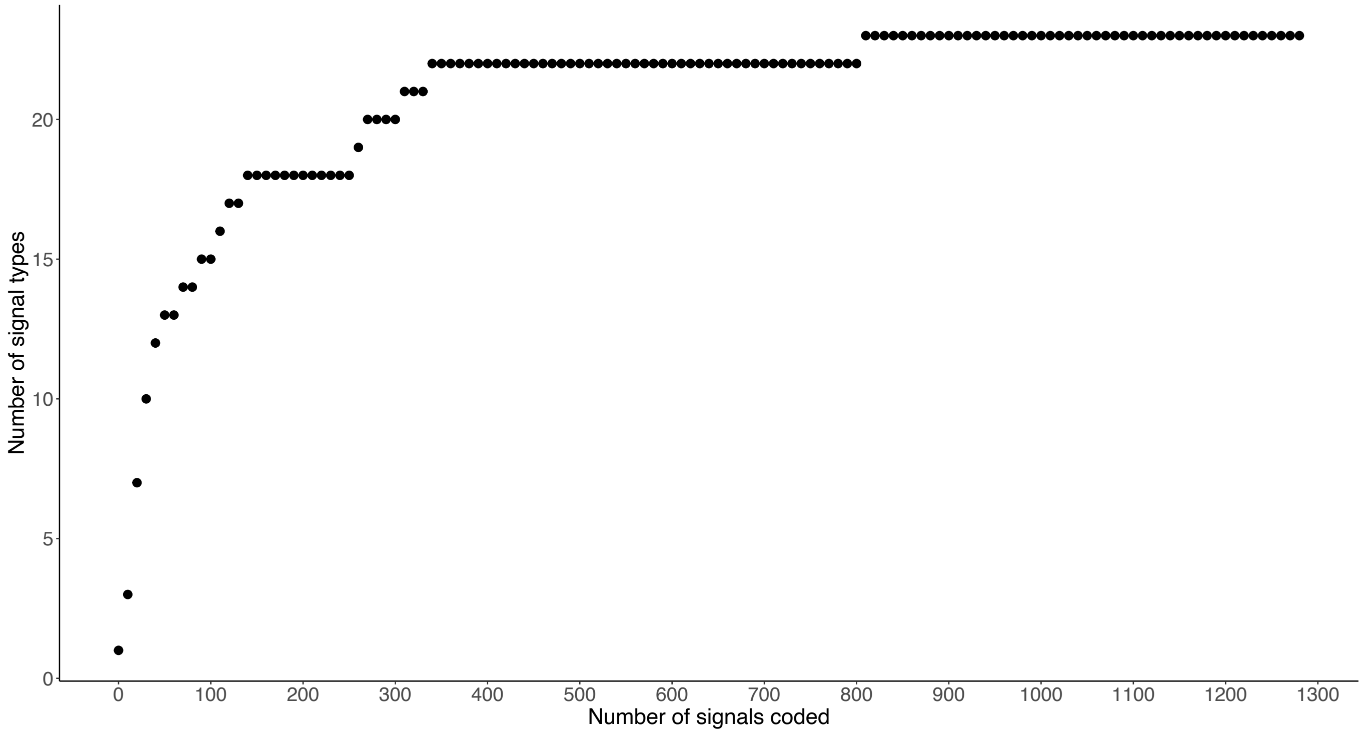

Supplementary Figure 1. Cumulative distribution of the repertoire of signal types used by the subjects during greeting. The cumulative number of signal types (*n*=23 signal types of which *n*=20 body act types, *n*=3 vocalisation types) coded is plotted against the total number of signal cases (*n*=1282). Asymptote is reached at around *n*=850 coded signal cases.

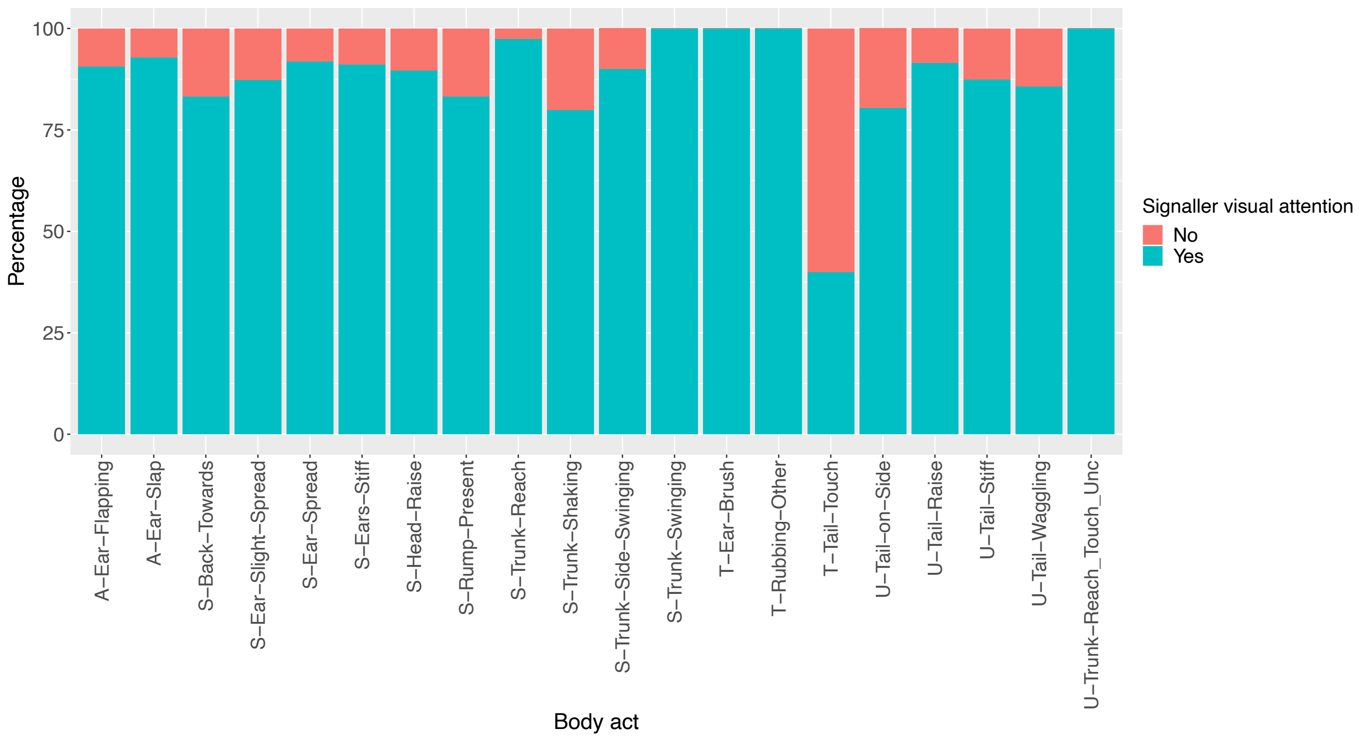

Supplementary Figure 2. Frequency of use of body act types where the signaller was visually attending or not the recipient at the onset of their production. “Yes” indicates that the signaller was attending the recipient; “No” indicates that the signaller was not attending the recipient. The letters preceding the body act names indicate the body act modality: A=Audible; S=Silent-visual; T=Tactile; U=Unknown (e.g., “A-Ear-Flapping”; S-Back-Towards; T-Ear-Brush; U-Tail-on-Side). *n*=1006 body acts.

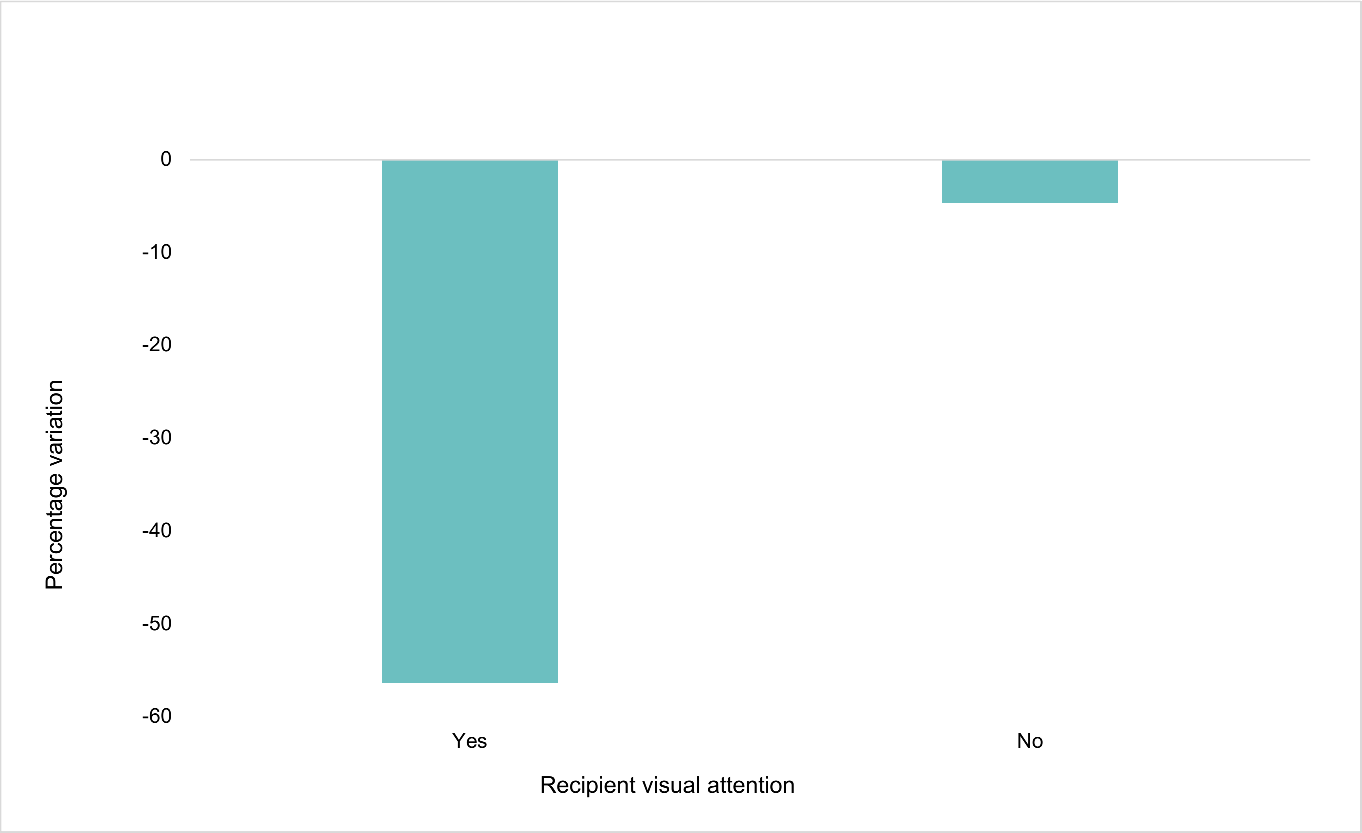

Supplementary Figure 3. Percentage variation in the use of Tail-on-Side, Tail-Raise, Tail-Stiff, and Tail-Waggling according to the recipient´s state of visual attention during greeting. Deviation above and below the 0 line show changes of frequency of use according to the recipient´s state of visual attention as compared to the normal distribution of silent-visual gestures. “Yes” indicates that the recipient was attending the body act; “No” indicates that the recipient was not attending the body act. *n*=543 body acts.

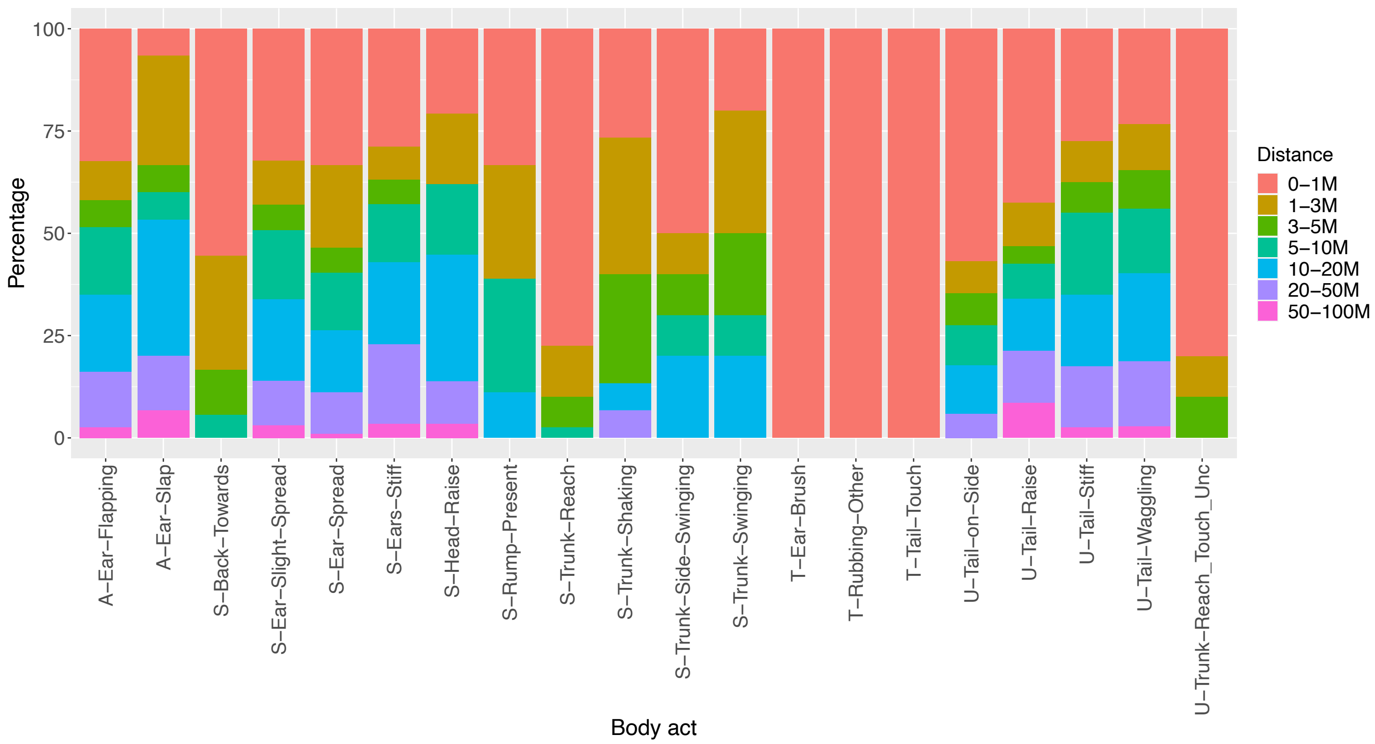

Supplementary Figure 4. Frequency of use of body act types at different distances (in m) during greeting. The letters preceding the body act names indicate the body act modality: A=Audible; S=Silent-visual; T=Tactile; U=Unknown (e.g., "A-Ear-Flapping"; S-Back-Towards; T-Ear-Brush; U-Tail-on-Side). *n*=1014 body acts.

Description of Supplementary Movie 1

The video starts with the male elephant Doma approaching the female elephant Kariba from the left. Kariba appears in the video at sec 01:20.

- **Supplementary Note 1 - Doma Body acts and Vocalisations:**

-Ear body acts: At sec 0:00 Doma is already flapping his ears (i.e., Ear-Flapping) until sec 02:09 where he stops. At sec 03:21 he resumes and at 09:23 Doma stops flapping his ears and spreads them (i.e., Ear-Spread). Then at sec 12:12 he pulls them back and at sec 14:09 he starts flapping the ears (i.e., Ear-Flapping) again until min 30:10.

-Tail body acts: At sec 0:00 Doma is walking with his tail stiff (i.e., Tail-Stiff). At sec 10:09 he raises his tail (i.e., Tail-Raise) and then at sec 13:05 he moves it to the left of his own body (i.e., Tail-on-Side) until sec 21:01, when he moves it to the other side (i.e., Tail-on-Side). The video ends before he relaxes his tail back down.

-Trunk body acts: At sec 12:12 Doma swings his trunk to the side (i.e., Trunk-Side-Swinging). At sec 14:07 Doma directs his trunk towards Kariba and touches her ear and then her mouth (i.e., Trunk-Reach_Touch).
-Limb body acts: At sec 12:23 Doma swings his left foot (i.e., Foot-Swinging).

-Vocalisations: Around sec 01:23 Doma starts rumbling. Around sec 09:03 he starts rumbling softly and then at 16:01 he rumbles again softly and at around 23:00 sec he rumbles more loudly.

- **Supplementary Note 2 - Kariba Body acts and Vocalisations:**

-Ear body acts: At sec 01:20 Kariba is approaching Doma holding her ears spread (i.e., Ear-Spread). At sec 02:20 she starts flapping her ears (i.e., Ear-Flapping) until sec 05:24, when she opens them holding them stiff (i.e., Ears-Stiff) while raising her head (i.e., Head-Raise). At sec 08:23 she starts flapping her ears again (i.e., Ear-Flapping). At sec 11:20 she stops flapping her hears and holds them stiff (i.e., Ears-Stiff) and then at sec 14:09 she spreads them (i.e., Ear-Spread). As her ears are spread Doma touches her left ear. At sec 18:14 she brushes her ear (i.e., Ear-Brush) on Doma´s trunk and face. At sec 20:08 she pulls her ears back and holds them stiff (i.e., Ears-Stiff). While she is behind Doma she flaps them a bit and then holds them stiff again as she walks in front.
Trunk body acts: At sec 16:07 she swings her trunk in front (i.e., Trunk-Swinging)
Rump body acts: At sec 14:32 she turns her rump back and walks back into Doma´s side (i.e., Back-Towards).
Tail body acts: From when she is visible Kariba waggles her tail (i.e., Tail-Waggling) until sec 06:05, when the tail is not visible to us anymore. At sec 10:23 her tail is held raised (i.e., Tail-Raise) and then at sec 11:19 starts waggling it (i.e., Tail-Waggling). From min 15:20 her tail is not visible anymore.
Mouth body acts: At sec 21:19 Kariba bites Doma´s trunk (i.e., Bite-Other).

-Vocalisations: At the start of the video Kariba is trumpeting while approaching Doma (not visible). Around sec 02:18 she trumpets again. Around sec 18:09 she starts rumbling loudly and again around sec 26:09.
