# Supplementary material for: Multimodal communication and audience directedness in the greeting behaviour of semi-captive African savannah elephants
Source: Commun Biol. 2024 May 9;7:472. doi: 10.1038/s42003-024-06133-5 (PMC11082179; doi:10.1038/s42003-024-06133-5)
Supplement: Supplementary file 3 — Description of Additional Supplementary Files [file 42003_2024_6133_MOESM3_ESM.pdf]

Description of Additional Supplementary Files

File name: Supplementary Data 1
Description: Calculations for “Nearest-neighbour” spatial proximity indexes (NN) of all elephant dyads.

File name: Supplementary Data 2
Description: Calculations for Percentage deviation of silent-visual, audible, and tactile body acts according to Recipient visual attention. The source behind Figure 4.

File name: Supplementary Data 3
Description: Calculations for Percentage deviation of potentially silent-visual tail body acts (i.e., Tail-on-Side, Tail-Raise, Tail-Stiff, Tail-Waggling) as compared to established silent-visual body acts according to Recipient visual attention. The source behind Supplementary Figure 3.

File name: Supplementary Data 4
Description: Results of Multiple Distinctive Collocation Analyses of the bigrams of vocalisation and body act types produced by the subjects during greeting.

File name: Supplementary Movie 1
Description: Video of example of a greeting event between a male and a female elephant.
