# Supplementary material for: Multimodal communication and audience directedness in the greeting behaviour of semi-captive African savannah elephants
Source: Commun Biol. 2024 May 9;7:472. doi: 10.1038/s42003-024-06133-5 (PMC11082179; doi:10.1038/s42003-024-06133-5)
Supplement: Supplementary file 9 — Reporting Summary [file 42003_2024_6133_MOESM9_ESM.pdf]

nature portfolio

Corresponding author(s): Vesta Eleuteri, Angela Stöger

Last updated by author(s): 10/03/2024

Reporting Summary

Nature Portfolio wishes to improve the reproducibility of the work that we publish. This form provides structure for consistency and transparency in reporting. For further information on Nature Portfolio policies, see our Editorial Policies and the Editorial Policy Checklist.

Statistics

For all statistical analyses, confirm that the following items are present in the figure legend, table legend, main text, or Methods section.

n/a	Confirmed	
☐	☒	The exact sample size (*n*) for each experimental group/condition, given as a discrete number and unit of measurement
☐	☒	A statement on whether measurements were taken from distinct samples or whether the same sample was measured repeatedly
☐	☒	The statistical test(s) used AND whether they are one- or two-sided<br>*Only common tests should be described solely by name; describe more complex techniques in the Methods section.*
☐	☒	A description of all covariates tested
☐	☒	A description of any assumptions or corrections, such as tests of normality and adjustment for multiple comparisons
☐	☒	A full description of the statistical parameters including central tendency (e.g. means) or other basic estimates (e.g. regression coefficient) AND variation (e.g. standard deviation) or associated estimates of uncertainty (e.g. confidence intervals)
☐	☒	For null hypothesis testing, the test statistic (e.g. *F*, *t*, *r*) with confidence intervals, effect sizes, degrees of freedom and *P* value noted<br>*Give P values as exact values whenever suitable.*
☐	☒	For Bayesian analysis, information on the choice of priors and Markov chain Monte Carlo settings
☐	☒	For hierarchical and complex designs, identification of the appropriate level for tests and full reporting of outcomes
☐	☒	Estimates of effect sizes (e.g. Cohen's *d*, Pearson's *r*), indicating how they were calculated

Our web collection on statistics for biologists contains articles on many of the points above.

Software and code

Policy information about availability of computer code

Data collection	No software was used for data collection.
Data analysis	We used R version 4.0.2 for statistical analyses.

For manuscripts utilizing custom algorithms or software that are central to the research but not yet described in published literature, software must be made available to editors and reviewers. We strongly encourage code deposition in a community repository (e.g. GitHub). See the Nature Portfolio guidelines for submitting code & software for further information.

Data

Policy information about availability of data

All manuscripts must include a data availability statement. This statement should provide the following information, where applicable:

- Accession codes, unique identifiers, or web links for publicly available datasets
- A description of any restrictions on data availability
- For clinical datasets or third party data, please ensure that the statement adheres to our policy

The datasets used to conduct statistical analyses are available on Github: The datasets used to conduct statistical analyses are available on Github96. Source data underlying Figure 3, Figure 5, and Supplementary Figure 1 can be found on Github96. Source data for Figure 4 and Supplementary Figure 3 can be found in Supplementary Calculations 2 and Supplementary Calculations 3.

Research involving human participants, their data, or biological material

Policy information about studies with human participants or human data. See also policy information about sex, gender (identity/presentation), and sexual orientation and race, ethnicity and racism.

Reporting on sex and gender	*Use the terms sex (biological attribute) and gender (shaped by social and cultural circumstances) carefully in order to avoid confusing both terms. Indicate if findings apply to only one sex or gender; describe whether sex and gender were considered in study design; whether sex and/or gender was determined based on self-reporting or assigned and methods used. Provide in the source data disaggregated sex and gender data, where this information has been collected, and if consent has been obtained for sharing of individual-level data; provide overall numbers in this Reporting Summary. Please state if this information has not been collected. Report sex- and gender-based analyses where performed, justify reasons for lack of sex- and gender-based analysis.*
Reporting on race, ethnicity, or other socially relevant groupings	*Please specify the socially constructed or socially relevant categorization variable(s) used in your manuscript and explain why they were used. Please note that such variables should not be used as proxies for other socially constructed/relevant variables (for example, race or ethnicity should not be used as a proxy for socioeconomic status). Provide clear definitions of the relevant terms used, how they were provided (by the participants/respondents, the researchers, or third parties), and the method(s) used to classify people into the different categories (e.g. self-report, census or administrative data, social media data, etc.) Please provide details about how you controlled for confounding variables in your analyses.*
Population characteristics	*Describe the covariate-relevant population characteristics of the human research participants (e.g. age, genotypic information, past and current diagnosis and treatment categories). If you filled out the behavioural & social sciences study design questions and have nothing to add here, write "See above."*
Recruitment	*Describe how participants were recruited. Outline any potential self-selection bias or other biases that may be present and how these are likely to impact results.*
Ethics oversight	*Identify the organization(s) that approved the study protocol.*

Note that full information on the approval of the study protocol must also be provided in the manuscript.

Field-specific reporting

Please select the one below that is the best fit for your research. If you are not sure, read the appropriate sections before making your selection.

☒ Life sciences ☐ Behavioural & social sciences ☐ Ecological, evolutionary & environmental sciences

For a reference copy of the document with all sections, see nature.com/documents/nr-reporting-summary-flat.pdf

Life sciences study design

All studies must disclose on these points even when the disclosure is negative.

Sample size	To ensure a sufficient sample size of greeting signals per individual we selected for the Separation-Reunion experiments 8 pairs of 6 elephants that showed strong social bonds (using the "Nearest-neighbour" spatial proximity index "NNAB") and that, thus, would be more likely to greet upon reunion. Since examination of the cumulative frequency of the signal types used by the elephants during greeting approaches asymptote, the sample size of the study is sufficient for our description of the greeting behaviour of our group of semi-captive African savannah elephants.
Data exclusions	1. To describe the repertoire of signals used by semi-captive elephants during greeting we included body act types and vocalization types used at least twice by at least two individuals, following previous research describing communicative repertoires in other species (e.g., apes: Hobaiter et al., 2011; Byrne et al., 2017). The visual acuity of elephants at large distances is still unclear. However, as elephants can see a 2.75 cm object at the tip of their 2 m trunk, we calculated that elephants would be able to detect a 2.5-3 m object, which represents the average height of our adult elephants, from around 180m away. Similarly, we calculated that they could detect a 1m object, which represents the minimum size of the body parts used to produce the body acts (e.g., tail, trunk, head, ear), from around 70m away. To be conservative, we excluded cases where the signaller produced a body act when the recipient was more than 100m away. We further excluded cases where we were unsure whether the signaller was aware of the presence of the recipient, for example, where the signaller was not oriented towards the recipient and/or did not subsequently approach the recipient.<br>2. To investigate whether elephants target their gesture modality at their audience during greeting, we restricted analyses to those individuals who contributed at least one body act in each modality and excluded cases where the visual state of attention of the recipient was unclear. Doing so resulted in a total of n=670 body act cases included in the analyses.<br>3. To explore whether elephants produce multicomponent combinations in specific combination types and orders during greeting, we conducted Multiple Distinctive Collocation Analyses. To fully capture how elephants combine vocalisations and body act types we conducted two separate MDCAs. The start times of consecutive signals produced with the same body part would depend on the end times of the preceding signal to them, affecting our ability to detect the order in which vocalisations and body acts are integrated into combinations. Thus, in MDCA1, if a signal A overlapped with more than one signal produced with the same body part, we included only the first of the overlapping signal. In MDCA2, we included the entire dataset of multiple co-occurring signals to explore the general frequency of co-occurrence of vocalisation and body act types without taking into consideration any pattern of ordering. The samples consisted in n=337 distinct bigrams of

vocalisations and body acts in the first MDCA and n=403 in the second MDCA.

Replication	Because our study consists in describing and exploring the use of signals and multicomponent combinations in semi-captive African savannah elephants, we did not require replication of any experiment design. The separation-reunion procedure was used to stimulate greeting behaviour in the subjects and not test to the subjects´ performance in specific experimental conditions.
Randomization	Because our study consists in describing and exploring the use of signals and multicomponent combinations in semi-captive African savannah elephants, we did not require any randomization procedure in our Separation-Reunion procedure. The procedure was used to stimulate greeting behaviour in the subjects and not test to the subjects´ performance in specific experimental conditions.
Blinding	Because our study consists in describing and exploring the use of signals and multicomponent combinations in semi-captive African savannah elephants, we did not require any blinding during data collection or analysis. We selected pairs of subjects that showed strong levels of social bond and that would, thus, be more likely to engage in greetings during reunions.

Reporting for specific materials, systems and methods

We require information from authors about some types of materials, experimental systems and methods used in many studies. Here, indicate whether each material, system or method listed is relevant to your study. If you are not sure if a list item applies to your research, read the appropriate section before selecting a response.

Materials & experimental systems

n/a	Involved in the study	
☒	☐	Antibodies
☒	☐	Eukaryotic cell lines
☒	☐	Palaeontology and archaeology
☐	☒	Animals and other organisms
☒	☐	Clinical data
☒	☐	Dual use research of concern
☒	☐	Plants

Methods

n/a	Involved in the study	
☒	☐	ChIP-seq
☒	☐	Flow cytometry
☒	☐	MRI-based neuroimaging

Animals and other research organisms

Policy information about studies involving animals; ARRIVE guidelines recommended for reporting animal research, and Sex and Gender in Research

Laboratory animals	The study did not involve laboratory animals.
Wild animals	The study did not involve wild animals. The study involved semi-captive African savannah elephants (Loxodonta africana) in the Jafuta Reserve in Victoria Falls, Zimbabwe. The semi-captive group consists of 9 elephants (four males and five females) who are engaged in non-invasive tourism interactions with humans. Their daily routine starts with them interacting with carers to receive any necessary medical care and for a 30-minute training session using cooperative teaching methods. Riding is not permitted and restraints are not used so that the elephants are free to end the interaction by moving away. During the day the elephants have unlimited access to the reserve and at night they are kept in small groups in adjacent bomas. After the study the elephants continued being engaged in tourism interactions and their daily routine.
Reporting on sex	We included both male and female elephants in our study as previous studies of elephant greetings provided descriptions of males and females performing different behaviours during greeting. Because our study group consisted in elephants under human care (i.e., semi-captive elephants), the identity and sex of the individuals was known. We explored whether the repertoire of greeting signals and multi-signal combinations varied between females and males. We found that females and males used the same repertoire of greeting signal types, except for the gesture type Rubbing-Other, which was observed only in males. Of the 1014 body acts coded, 562 were produced by females and 452 by males, while of the 268 vocalisations coded, 155 by females and 113 by males. We found that female elephants use more frequently ear-flapping gestures and rumble vocalisations in combination when greeting each other, confirming previous descriptions of female elephant greetings.
Field-collected samples	The study did not involve samples collected in the field.
Ethics oversight	Ethical approval for the study was given by the Faculty of Life Sciences of the University of Vienna (Ethical Approval No.2021-021).

Note that full information on the approval of the study protocol must also be provided in the manuscript.
